# Navigating challenges in diagnosing acquired hemophilia A: A case report from Syria

**DOI:** 10.1002/ccr3.8811

**Published:** 2024-04-24

**Authors:** Oubai Nayouf, Miriam Laflouf, Ibrahim Hamdan, Ibrahim Alghazawi, Omar Aldairi, Ameen Sulaiman

**Affiliations:** ^1^ Faculty of Medicine Damascus University Damascus Syria; ^2^ Department of Hematology, Faculty of Medicine Damascus University Damascus Syria; ^3^ Department of Internal Medicine, Hematology, Al‐Mouwasat Hospital Damascus University Damascus Syria

**Keywords:** acquired hemophilia, case report, FVIII, postpartum period

## Abstract

Acquired hemophilia A is a rare bleeding disorder. Rapid diagnosis with prolonged aPTT and low FVIII, and immediate use of bypassing agents and steroids are crucial for better outcomes, highlighting the importance of early recognition and management.

## Graphical abstract

Early diagnosis and intervention are crucial for better outcomes in acquired hemophilia A.

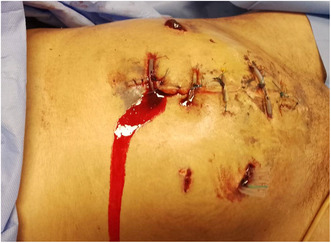

## 1 INTRODUCTION

Acquired hemophilia A (AHA) is an uncommon condition that develops due to the presence of autoantibodies against coagulation factor VIII (FVIII). Among the various coagulation factors, FVIII is the most frequently targeted by autoantibodies, with an estimated annual incidence rate of roughly 1–1.5 cases per million individuals.^1^ AHA can manifest at any age, with the average age of onset being 70 years old. Approximately 50% of AHA cases are idiopathic. The other causes are related to autoimmune disorders or pregnancy, particularly in younger patients.^2^ The most common type of bleeding seen in these patients is subcutaneous, which affects about 80% of cases. This is followed by muscular, gastrointestinal, genitourinary, and retroperitoneal hemorrhages in descending order of occurrence. When an unexpectedly prolonged aPTT is observed, AHA should be suspected. Further diagnostic procedures, such as the mixing test and the Bethesda assay (BA), are necessary to confirm AHA. The management of AHA includes stabilizing the patient's hemostatic condition and the administration of treatments like steroids, cyclophosphamide, and rituximab, which are immunosuppressive in nature.^3^ We present the case of an 18‐year‐old married female who experienced delayed postpartum bleeding at the age of 15 without undergoing a proper evaluation. Three years later, she presented with acute abdominal pain and was thereafter diagnosed with AHA after appropriate investigations.

## 2 TIMELINE

The timeline of the patient's symptoms, examinations, and treatment is summarized in Table 1.

**TABLE 1:** The timeline of the patient's symptoms, examinations, treatments, and follow‐up.

DD/MM/YYYY	Chief complaint	Investigations	Treatment and follow‐up
2020_3 years earlier	Gynecology and obstetrics department/labor pains	Physical examination: active labor	Vaginal delivery
Five days postpartum	Gynecology and obstetrics department/postpartum hemorrhage, epistaxis, and ecchymoses	Physical examination: postpartum hemorrhage, epistaxis, and ecchymosis	D&C, whole blood transfusion for 2 months
13/11/2023_20 days before admission	ED/acute abdominal pain, malaise, fatigue, and vomiting after eating	Physical examination: generalized abdominal tenderness Blood tests: Hb 3.5 g/dL	Whole blood transfusion
23/11/2023_10 days before admission	Central hospital ED/hypovolemic shock along with abdominal distention	Ultrasonography: intra‐abdominal free fluid alongside a left ovarian cyst Blood tests: aPTT 92.6, PT 18.9, INR 1.38 Mixing test: prolonged aPTT, normal PT, and INR	Emergency exploratory laparotomy Histopathology: right ovarian torsion cyst and a left ovarian hemorrhagic cyst
28/11/2023_5 days before admission	Central hospital ED/hypovolemic shock along with abdominal distention	Ultrasonography: intra‐abdominal free fluid alongside a left ovarian	Exploratory laparotomy
3/12/2023_on admission	Hematology department/multiple ecchymoses, and active bleeding from the drain	Vital signs: heart rate 130 bpm, blood pressure 130/80 mmHg, oxygen saturation 99%, temperature 38°C Physical examination: severe pallor, multiple ecchymoses, and active bleeding from the drain Mixing test results: Abnormal FVIII inhibitor titer 145 (BU/mL) FVIII activity: 0.1%	FFP, rFVIIa, FEIBA, prednisone, and azathioprine
3/1/2024_month after admission	Hematology department/follow‐up	Physical examination: favorable progress, the hemorrhagic drainage diminished, the bleeding was completely controlled	The same treatment
7/1/2024_5 days before discharge	Hematology department/follow‐up	Vital signs: normal Physical examination: pallor, no ecchymoses	The administration of FFP and rFVIIa was discontinued
12/1/2024_on discharge	Hematology department/follow‐up	Vital signs: normal Physical examination: the drain infection was successfully treated, pallor, no ecchymoses Blood tests: Hb 9 g/dL, aPTT 48.9 s	Prednisone. Azathioprine was stopped
15/1/2024_forty 3 days after admission	Hematology department/ follow‐up	No complications Blood tests: aPTT 50, PT 13.8, INR 1.02, Hb 10.8 g/dL	Prednisone
11/3/2024_2 months after discharge	Hematology department/follow‐up	No complications Blood tests: aPTT 31, PT 17, INR 1.38, PLT 310, HCT 31, Hb 9.73, RBCs 2.7, MCV 115, MCH 34.56, MCHC 30, RDW 17%	Prednisone B9, B12 supplements

Abbreviations: aPTT, activated partial thromboplastin time; bpm, beats per minute; BU/mL, bethesda units per milliliter; D&C, dilation and curettage; ED, emergency department; FEIBA, factor eight inhibitor bypassing activity; FFP, fresh frozen plasma; Hb, hemoglobin; HCT, hematocrit; INR, international normalized ratio; MCH, mean corpuscular hemoglobin; MCHC, mean corpuscular hemoglobin concentration; MCV, mean corpuscular volume; PLT, platelet count; PT, prothrombin time; RBCs, red blood cells count; RDW, red cell distribution width; rFVIIa, recombinant factor VIIa.

## 3 CASE HISTORY/EXAMINATION

An 18‐year‐old woman was referred to the Department of Hematology for further investigation due to a 3‐week history of severe internal bleeding. The patient had regular menstrual cycles with no documented history of excessive menstrual bleeding or noteworthy personal or familial background of blood hemostasis disorders.

**FIGURE 1:**
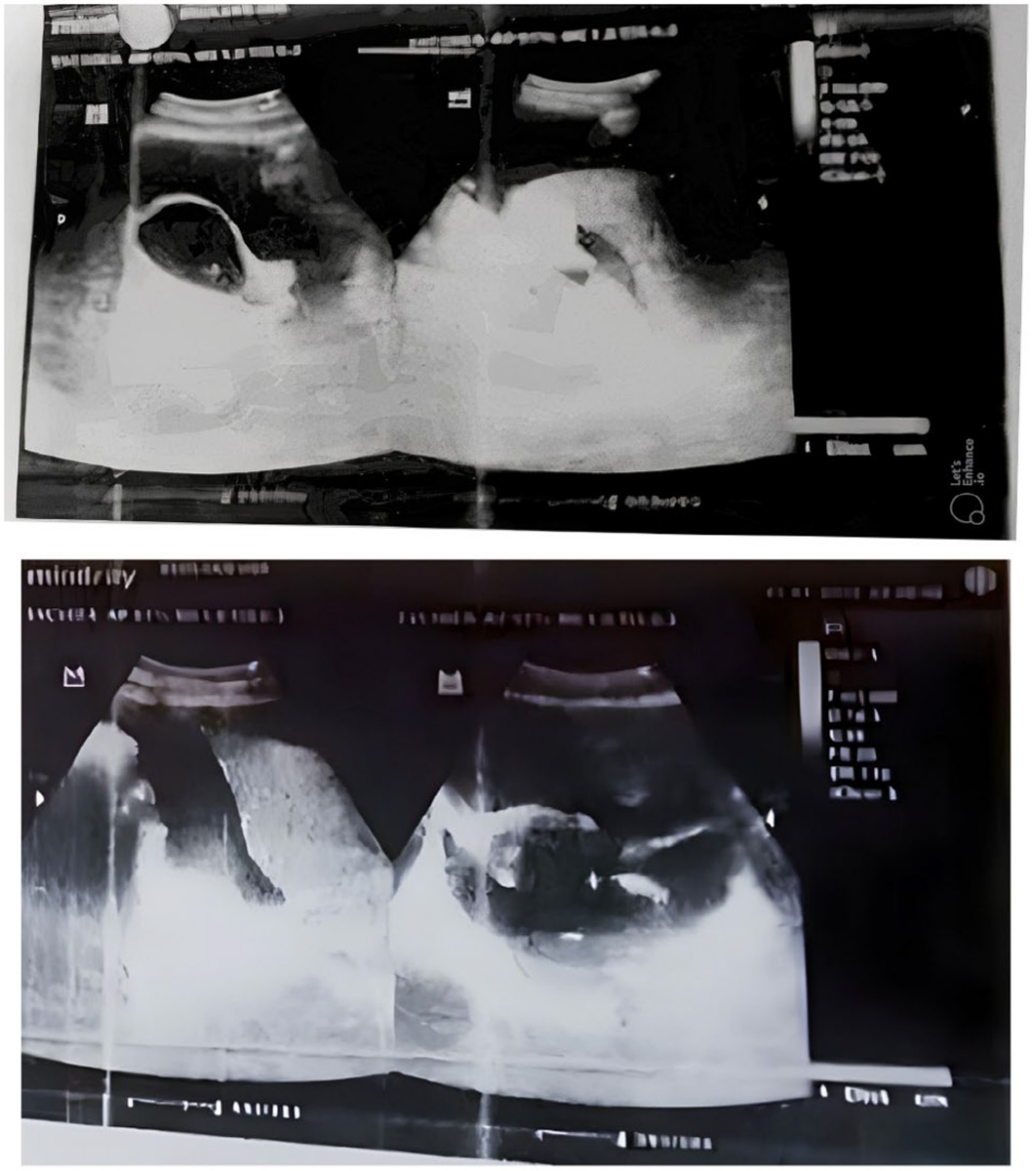
An abdominal echocardiogram showing free turbid fluid in the abdominal cavity.

Three years earlier, she had a successful and uncomplicated full‐term vaginal delivery during her first pregnancy. However, 5 days later, she experienced postpartum hemorrhage, epistaxis, and ecchymoses on her arm. These symptoms spontaneously resolved following a dilation and curettage (D&C) procedure and 2 months of whole blood transfusion. Twenty days before her admission to our department, she presented to the emergency department (ED) with acute abdominal pain, malaise, fatigue, and vomiting after eating. Physical examination revealed generalized abdominal tenderness, and emergency blood tests indicated a low hemoglobin (Hb) level (3.5 g/dL; normal range 12–15 g/dL). She received whole blood transfusion; however, her symptoms worsened, leading to her referral to the central hospital. In the ED, initial assessment revealed signs of hypovolemic shock along with abdominal distention. Further examinations began with ultrasonography, which revealed intra‐abdominal free fluid alongside a left ovarian cyst (Figure 1).

With the family's consent, an emergency exploratory laparotomy was conducted, during which a heavy amount of blood and clots was evacuated. Furthermore, a hemorrhagic cyst was excised from the left ovary while another cyst was identified on the right ovary. Excisional Biopsies were performed on both cysts, and the histopathology showed a right ovarian torsion cyst and a hemorrhagic cyst in the left ovary. Ovarian drilling was carried out, achieving hemostasis before abdominal closure and the placement of a drain. Coagulation parameters showed the following (Table 1): an isolated prolonged aPTT (92.6 s; normal range 25–36 s), which remained prolonged even after mixing in the inhibitor screen, while there was a relatively slight prolongation in the prothrombin time (PT) (18.9 s; normal range 12.3–14 s) and an elevated international normalized ratio (INR) (1.38; normal range 0.8–1.2). However, both the PT and INR were corrected after mixing.

**FIGURE 2:**
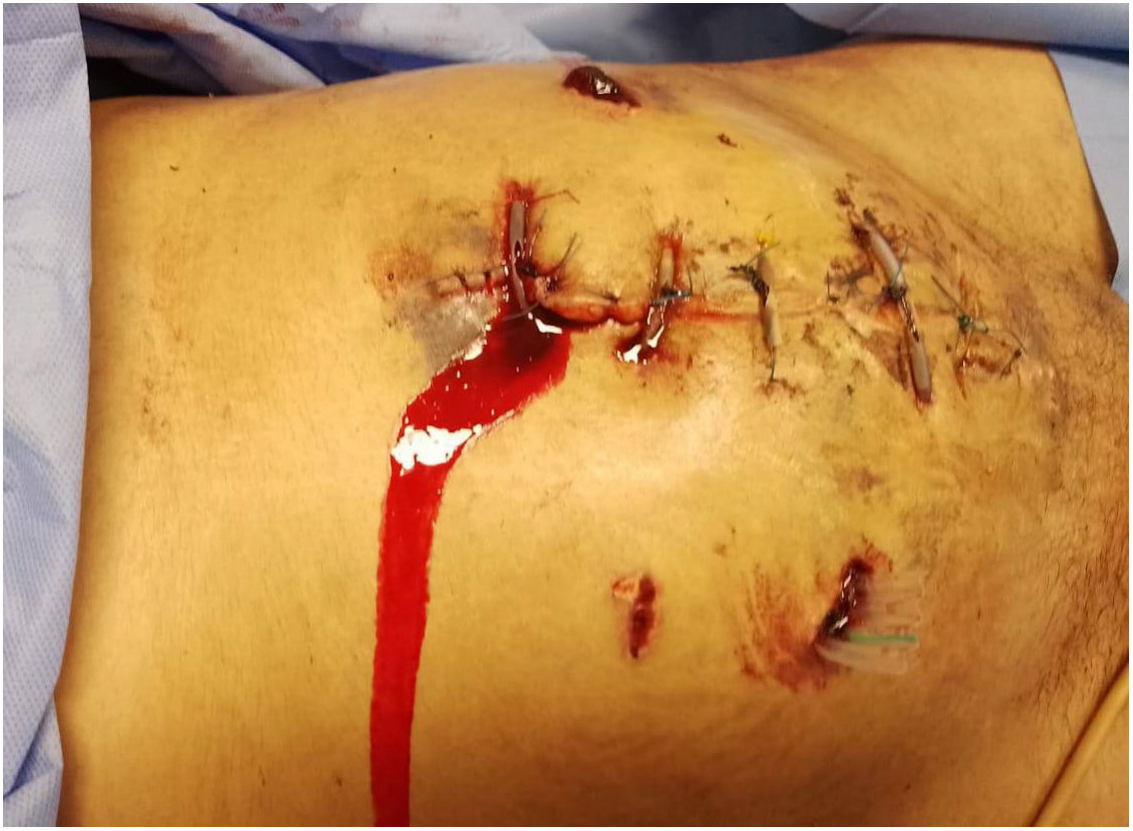
Active bleeding from the drain and surgical sutures.

In the subsequent days, the patient's condition deteriorated, exhibiting similar findings to those that prompted her first surgery, along with persistent coagulopathy. As a result, a second exploratory laparotomy was performed, suspecting disseminated intravascular coagulation (DIC). Massive bleeding with clots was once again observed, and the procedure was completed following the same approach as the previous intervention. Subsequently, the patient was transferred and admitted to our department. On admission, the patient's vital signs were recorded as follows: an elevated heart rate (130 beats per minute (bpm); normal range 60–100 bpm), with heightened blood pressure (130/80 mmHg; normal range 90–120 mmHg systolic, 60–80 mmHg diastolic), a normal oxygen saturation (99%; normal range 95%–100%), and a low‐grade fever (38°C; normal range 36.5–37.5°C), indicating a surgical drain infection. The physical examination revealed severe pallor, multiple ecchymoses on her upper limbs and both feet, as well as active bleeding from the drain and surgical sutures of the last operation (Figure 2).

## 4 DIFFERENTIAL DIAGNOSIS, INVESTIGATIONS, AND TREATMENT

The presence of a prolonged aPTT (92.6 s) suggested an existing coagulopathy. Considering the patient's bleeding history and the significant aPTT prolongation, AHA was suspected. A mixing test was subsequently conducted to distinguish between a deficiency in clotting factors and the presence of inhibitors. The sustained aPTT prolongation after the mixing test indicated that inhibitors were indeed present. Differential diagnosis included Lupus anticoagulant (LA) and anticoagulant‐induced coagulopathy. However, the absence of anticoagulant therapy in the patient's history lessened the chance of a drug‐induced issue. Instead of the dilute Russell's viper venom time (dRVVT) for LA, an Antinuclear Antibodies (ANA) test was negative, ruling out SLE and decreasing the suspicion of LA as the cause. Thus, the suspicion of AHA increased, and FVIII activity assay was performed which was notably reduced (0.1%; normal range 50%–150%). To further clarify the situation, an evaluation of the FVIII inhibitor titer was conducted, showing a remarkably elevated value of Bethesda Units per milliliter (145 BU/mL; normal level <0.6 BU/mL), confirming the presence of inhibitors and leading to the diagnosis of AHA. The patient at hand, presented with a severe case of AHA and was treated with fresh frozen plasma (FFP) at a dose of 20 mg/kg to provide replacement clotting factors and stabilize coagulation; this FFP treatment was carefully titrated based on aPTT and bleeding follow‐up to ensure adequate compensation. To manage bleeding, the patient received recombinant factor VIIa (rFVIIa) at a dosage of 90 mcg/kg, tailored to her response. Initially, rFVIIa was given every 4 h for 2 days, then every 6 h for the next 2 days, and every 8 h for the subsequent 3 days. Following this intensive initial week, the treatment was extended at suitable intervals for an additional month, ceasing 5 days before discharge. rFVIIa is essential as a bypassing agent, enabling clot formation without functional FVIII. Along with rFVIIa, the patient received Factor Eight Inhibitor Bypassing Activity (FEIBA) at a dose of 50 U/kg every 12 h for a duration of 3 days, to bypass the inhibited FVIII and mitigate the bleeding risk, albeit with a careful eye on the associated thrombotic risk. For immunosuppression, prednisone was initiated at a dose of 1 mg/kg/day orally (PO), with the total daily dose divided into 40 mg in the morning and 20 mg at noon, to suppress the production of autoantibodies against FVIII, contributing to the AHA. Concomitantly, azathioprine was started at 2 mg/kg/day PO, with an initial dose of 120 mg daily for 10 days, then reduced to 100 mg daily for a month, to further modulate the immune response and decrease antibody production. The treatment aimed to address both the bleeding episodes and underlying autoimmunity, with vigilant monitoring for thrombosis, hyperglycemia, and myelosuppression due to the potential adverse effects of the prescribed therapies.

## 5 OUTCOME AND FOLLOW‐UP

Within a month of admission, the patient showed favorable progress, and the hemorrhagic drainage diminished. As the bleeding was completely controlled, specifically 5 days before the discharge, we discontinued the administration of FFP and rFVIIa. On the day of discharge, azathioprine was stopped, the patient's vital signs were normal and no fever was documented. The drain infection was successfully treated, and while the patient still exhibited pallor upon physical examination, no ecchymoses were found. Laboratory tests revealed an Hb level of 9 g/dL, and an aPTT of 48.9 s. The rest of the coagulation profile was within the reference range. After a 43‐day follow‐up since admission, the patient didn't experience any complications, and her blood parameters gradually improved, as shown in Table 1. Two months post‐discharge, the patient has maintained good health without any new bleeding episodes. The ongoing management includes a gradually reduced dose of prednisone, currently at 15 mg/day. Additionally, she has commenced supplementation with vitamins B12 and B9 to treat her macrocytic anemia, as recent blood work has suggested (Table 1).

## 6 DISCUSSION

AHA is a seldom‐occurring bleeding disorder, with an incidence rate ranging from one to six cases per million individuals annually. The pathophysiology of AHA involves a breakdown in immune tolerance, leading to the production of autoantibodies against FVIII, an essential blood‐clotting protein. This loss of tolerance may be due to dysfunction in regulatory T cells or specific genetic factors that cause abnormal recognition of FVIII by the immune system, thereby triggering an autoimmune response that inhibits blood clotting. Registry issues, lack of awareness, and anticoagulant use in seniors contribute to disease underreporting, and its swift onset can delay diagnosis.^3^ AHA exhibits two age peaks: one in older males (>65 years) and another in younger females (20–40 years), primarily associated with postpartum cases.^4^ In our case, the patient is only 18 years old, and was 15 when she experienced the postpartum bleeding, and to our knowledge, this disorder has not been reported at this age in Syria before. AHA is clinically characterized by the abrupt emergence of bleeding without warning, affecting individuals without any previous personal or familial history of bleeding disorders. The severity of AHA‐related bleeding can vary widely from mild to life‐threatening, often presenting as extensive subcutaneous bruises, muscle hematomas, and mucosal bleeds, along with prolonged postoperative bleeding.^5^ AHA that is linked to pregnancy is most commonly seen during the postpartum phase, typically from 1 to 4 months after delivery.^3^ In patients with new abnormal bleeding, particularly the elderly, peripartum, or postpartum women, an isolated prolonged aPTT with normal PT warrants consideration of AHA.^6^ A retrospective study demonstrated that when the diagnosis was postponed for over a month, it resulted in an escalated demand for hemostatic factors and extended durations of active bleeding.^7^ Registry issues, lack of awareness, and anticoagulant use in seniors contribute to disease underreporting, and its swift onset can delay diagnosis.^3^ Regarding our patient, she experienced severe post‐pregnancy bleeding at age 15, which spontaneously stopped after a D&C procedure and 2 months of blood transfusions. Recently, she was admitted to our department following an ED visit for abdominal pain, malaise, fatigue, vomiting, and amenorrhea. An emergency exploratory laparotomy was performed, and a left ovarian bleeding cyst, later identified as a hemorrhagic cyst, was removed. Upon admission, the patient's coagulation profile displayed a prolonged aPTT (92.6 s), slightly prolonged PT (18.9 s), and elevated INR (1.38). These irregularities, coupled with her bleeding history, raised suspicion of a bleeding disorder, potentially AHA. To confirm, a targeted diagnostic workup was initiated. Differentiating prolonged aPTT involves targeted tests: factor deficiencies call for specific assays, LA by dRVVT and mixing studies, heparin effects by anti‐FXa assays, and direct inhibitors by appropriate anti‐FXa and TT assays. Vitamin K antagonist via PT and INR.^8^ In our case, we immediately proceeded to a mixing study to identify the presence of inhibitors, bypassing further tests to rule out other causes of aPTT prolongation. This standard diagnostic method mixes the patient's plasma with normal plasma in a 1:1 ratio. The aPTT is measured immediately and after 2 h at 37°C. An initial normalization followed by a prolongation of aPTT indicates an inhibitor presence, possibly FVIII in AHA.^8^ Other conditions can influence mixing test outcomes, mimicking the presence of circulating inhibitors, such as LA, heparins, and direct anticoagulants which need to be excluded.^6^ In our case, aPTT significantly prolonged after a 2‐h incubation period. Notably, the patient had no history of anticoagulant medication, which helped to exclude these agents as a cause of the prolonged aPTT. ANA test was performed to rule out SLE and returned negative, eliminating SLE as an underlying factor. Beyond the ANA test, no additional examinations were carried out to rule out other potential causes for the abnormal mixing study results. With these results, the suspicion of AHA became more pronounced. AHA diagnosis is confirmed through decreased FVIII:C activity and inhibitor measurement using BA. Bethesda units classify AHA patients with low (<5 BU/mL) or high (>5 BU/mL) inhibitor titers based on residual FVIII:C activity after mixing patient plasma with normal plasma.^8^ FVIII activity assay was performed showing remarkable reduction (0.1%), along with BA with 145 BU/ml confirmed AHA diagnosis. Approximately 50% of AHA cases are labeled as idiopathic due to the absence of identified underlying diseases. The remaining cases are associated with autoimmune disorders (such as SLE, rheumatoid arthritis (RA), and thyroid disorders), hematologic or solid cancers, infections, dermatological diseases, drugs, and pregnancy.^5^ Autoimmune diseases often lead to the development of autoantibodies against FVIII, particularly in conditions like RA and SLE. These conditions are typically associated with high titer inhibitors and a poor response to steroid treatment. In regards to AHA and pregnancy, AHA usually occurs postpartum, with abnormal bleeding being a common symptom. Rare instances of these inhibitors have been reported during pregnancy, often leading to severe complications.^3^ In this particular case, only SLE and RA were ruled out through ANA and RF tests, respectively, both yielding negative results. Other potential causes for AHA, such as additional autoimmune diseases, malignancies, and infections, were not investigated due to financial constraints. The medical team confirmed the diagnosis of AHA 20 days after the last emergency, which was caused by a bleeding cyst. This was interpreted as an undiagnosed complication of postpartum AHA following her first pregnancy. In AHA management there are two main aspects, the first is bleeding control; using bypassing agents like rFVIIa^8^ (recommended dose: 70–90 mcg/kg every 2–3 h until hemostasis achieved),^6^ and activated prothrombin complex concentrates (APCC) (FEIBA, Baxalta, Bannockburn, IL, USA) as a primary choice,^8^ (recommended dose: 50–100 U/kg every 8–12 h up to a maximum of 200 U/kg/day).^6^ Tiede et al.^9^ recommend that for patients with AHA who are experiencing clinically serious bleeding, hemostatic treatment should be initiated, irrespective of their inhibitor titer and residual factor VIII activity. The other goal is inhibiting or eliminating the cellular clone responsible for autoantibody synthesis, by using immunosuppressive therapy (IST) which includes the use of corticosteroids alone like prednisone^8^ (recommended dose:1 mg/kg PO daily),^6^ as first‐line therapy, or combined with cytotoxic agents, such as cyclophosphamide^8^ (recommended dose: 1–2 mg/kg PO daily),^6^ or rituximab^8^ (recommended dose: 375 mg/m^2^ IV weekly × 4).^6^ Tiede et al.^9^ suggest a first‐line therapy for patients with FVIII levels below 1 IU/dL or an inhibitor titer above 20 BU, which involves a combination of corticosteroids with either rituximab or a cytotoxic agent. Our patient was found to have significant inhibitor titers of 145 BU/mL. Upon conducting a laparotomy, we discovered recurrent episodes of substantial bleeding. To manage these episodes and maintain clotting activity, we administered rFVIIa at a dosage of 90 mcg/kg and FEIBA at a dose of 50 U/kg. Considering her elevated inhibitor titers, we also initiated an associated IST regimen, administering prednisone at 1 mg/kg/day orally and azathioprine at 2 mg/kg/day orally. These dosages align with current literature recommendations. Patients receiving bypassing agents are at increased risk of DIC, and arterial and venous thromboembolism, especially in older patients and those with underlying conditions such as previous thrombosis, malignancy, or other thrombotic risk factors.^6^ Corticosteroids, despite their effectiveness, can lead to immunosuppression, mood changes, and elevated blood glucose, potentially requiring Pneumocystis pneumonia prophylaxis during long‐term use. Conversely, cyclophosphamide, while potent, may increase the risk of secondary cancers like bladder cancer and myelodysplastic syndrome with prolonged use. It also necessitates close monitoring of complete blood counts due to its myelosuppressive nature and can cause renal and gonadal toxicity, emphasizing the need for adequate hydration and careful use. On the other hand, rituximab increases the risk of viral infections and can lead to hepatitis B reactivation.^1^ AHA has a relapse rate of 7.1%–20%, with relapse typically occurring in 7.5 months. Treatment‐wise, relapse rates stand at 18% for steroids, 12% with additional cyclophosphamide, and 4% with rituximab regimens. Postpartum AHA can see relapse rates up to 29% around 8 months.^3^ Furthermore, AHA is linked with a pronounced mortality rate, between 6.7% and 38%, with infection, likely due to required immunosuppression, being the primary cause of death.^1^ Regular follow‐ups are recommended after eradication therapy, with serial aPTT measurements and monthly monitoring of FVIII during the first 6 months. In the subsequent months, follow‐ups should be conducted every 2–3 months for up to 1 year, and then every 6 months thereafter.^9^ Kruse‐Jarres et al.^6^ encourage that, during IST, both inhibitor titer and FVIII activity should be monitored on a weekly basis, at the minimum. The patient at hand was followed up clinically, and laboratory by measuring aPTT values (Table 1). Her general condition improved significantly, and no complications developed during this period. Ultimately, optimal care for AHA requires the integrated efforts of critical specialties, including hematology, obstetrics, and immunology, to ensure a holistic approach to both treatment and overall patient well‐being. In conclusion, the timely recognition of AHA in spontaneous bleeding events is essential. This case report highlights the necessity for heightened vigilance and early intervention among clinicians dealing with AHA, a condition that can stem from a myriad of sources such as autoimmune disorders, malignancies, infections, dermatological conditions, medications, and pregnancy. In this case, despite our comprehensive efforts to identify the cause, budgetary limitations prevented a full exploration of all potential triggers. Moreover, while it is recommended that, during IST, inhibitor titer and FVIII activity should be monitored at least weekly, our patient's follow‐up was constrained to clinical assessments and aPTT measurements. It is also noteworthy that the diagnosis of AHA was not established during the initial postpartum bleeding but was only identified 3 years later following a ruptured hemorrhagic cyst, leading us to retrospectively consider the earlier episode as potentially related to AHA. Despite these limitations, this report contributes important insights into AHA, underscoring the necessity for broader research to enhance our approaches to diagnosing and treating this condition.

## AUTHOR CONTRIBUTIONS

**Oubai Nayouf:** Project administration; validation; writing – original draft; writing – review and editing. **Miriam Laflouf:** Resources; validation; writing – original draft; writing – review and editing. **Ibrahim Hamdan:** Writing – original draft; writing – review and editing. **Ibrahim Alghazawi:** Writing – original draft. **Omar Aldairi:** Writing – original draft. **Ameen Sulaiman:** Supervision; validation; writing – review and editing.

## FUNDING INFORMATION

Not applicable.

## CONFLICT OF INTEREST STATEMENT

The authors declare no conflict of interest.

## CONSENT

Written informed consent was obtained from the patient to publish this report in accordance with the journal's patient consent policy.

## Data Availability

Data sharing is not applicable to this article as no new data were created or analyzed in this study.
